# Effect of Statin Lipophilicity on the Proliferation of Hepatocellular Carcinoma Cells

**DOI:** 10.3390/biology13060455

**Published:** 2024-06-19

**Authors:** Goda Glebavičiūtė, Akshay Kumar Vijaya, Giulio Preta

**Affiliations:** Institute of Biochemistry, Life Science Center, Vilnius University, LT-10257 Vilnius, Lithuania; goda.glebaviciute@gmc.stud.vu.lt (G.G.); akshay.vijaya@gmc.vu.lt (A.K.V.)

**Keywords:** statins, lipophilicity, cytotoxicity, organic anion transporters

## Abstract

**Simple Summary:**

This study examines how statins, which are drugs commonly used to lower cholesterol, affect the growth of liver cancer cells. Statins can be either lipophilic (lipid-soluble) or hydrophilic (water-soluble), and this characteristic influences how they enter cells. In our research, we compared the effects of lipophilic simvastatin and hydrophilic pravastatin. We found that simvastatin significantly reduces cancer cell growth and increases cell death depending on the dosage and duration of treatment. In contrast, pravastatin, due to its limited uptake, has a minimal impact on cancer cells. These findings suggest that the type of statin used could be crucial in cancer treatment, potentially offering better outcomes for patients with liver cancer.

**Abstract:**

The HMG-CoA reductase inhibitors, statins, are drugs used globally for lowering the level of cholesterol in the blood. Different clinical studies of statins in cancer patients have indicated a decrease in cancer mortality, particularly in patients using lipophilic statins compared to those on hydrophilic statins. In this paper, we selected two structurally different statins (simvastatin and pravastatin) with different lipophilicities and investigated their effects on the proliferation and apoptosis of hepatocellular carcinoma cells. Lipophilic simvastatin highly influences cancer cell growth and survival in a time- and concentration-dependent manner, while pravastatin, due to its hydrophilic structure and limited cellular uptake, showed minimal cytotoxic effects.

## 1. Introduction

Statins, also known as HMG-CoA reductase inhibitors, are a class of drugs commonly prescribed to lower low-density lipoprotein (LDL) cholesterol in the blood. They are typically classified based on their lipophilicity, which is an important factor in a drug’s ability to penetrate cell membranes and tissues [1,2]. LogP is a measure widely used to describe the lipophilicity of a compound and is calculated as the logarithm of the partition coefficient, which is the ratio of a compound’s concentration in a non-polar solvent (usually octanol) to its concentration in a polar solvent (usually water). In the context of statins, logP is often used to predict a statin’s ability to interact with and cross cell membranes, with a high logP value suggesting a greater lipophilicity and potential for better penetration of lipid-rich environments [3]. Beyond the cholesterol-lowering effects, statins also possess several pleiotropic or cholesterol-independent effects, including anti-proliferative, immunomodulatory and antioxidant properties [4,5,6]. Among the identified mechanisms behind statins’ pleiotropy, there is also the ability to change membrane bilayer properties due to their amphiphilic nature [7,8,9]. In this manuscript, we selected simvastatin with a logP of 3.79 (highly lipophilic) and pravastatin with a logP of 1.65 (highly hydrophilic) and evaluated their effects on proliferation and apoptosis of the hepatocellular carcinoma (HCC) cell lines HepG2, the most commonly used experimental model for in vitro liver cancer research [10,11]. We observed that simvastatin as a lipophilic statin showed a more pronounced cytotoxic effect compared to hydrophilic pravastatin. The lower cytotoxic potential of pravastatin is probably related to its reduced accumulation in liver cells due to its hydrophilic structure and the necessity to enter via active transport using organic anion transporters (OATP1B1, OATP1B3, OATP2B1) poorly expressed in our tumor cell line [12,13]. These results were validated using different assays based on mitochondrial respiration (MTT) and on membrane integrity (LDH assay) and evaluations of nuclear morphology (DAPI staining). This was a necessary precaution since several studies show that statins can affect mitochondria directly via inhibition of respiratory chain complexes or indirectly via lowering of ubiquinone levels, a factor that can lead to misinterpretation of the assays based merely on mitochondrial respiration [14,15]. To further support our observations, we selected additional immortalized cell lines (A431, HeLa, HCT116, PC-3) with different expressions of organic anion transporters and included atorvastatin, the statin with the highest logP (5.39), in the analysis. Once again, cytotoxic activity was connected to lipophilicity, with atorvastatin showing more cytotoxic effects in all tested cells. In general, our findings validate the significance of statins’ physicochemical attributes, such as lipophilicity, in shaping their pleiotropic effects. This underlines the importance of considering these factors when selecting statins as adjuvants in cancer therapy.

## 2. Material and Methods

### 2.1. Cell Culture

The following immortalized cell lines were used in this study: HepG2 (hepatocellular carcinoma), A431 (human epidermoid carcinoma), HeLa (cervical carcinoma), HCT116 (colorectal carcinoma), and PC-3 (prostate carcinoma). All cell lines were maintained in DMEM + GlutaMAX medium supplemented with 10% fetal bovine serum (FBS), 100 IU/mL penicillin and 100 μg/mL streptomycin (all from Thermo Fisher Scientific, Waltham, MA, USA) in a humified incubator at 37 °C and 5% CO_2_. Cells were regularly checked for mycoplasma using a Mycoplasma detection kit (InvivoGen, Toulouse, France).

### 2.2. Statins

Simvastatin, atorvastatin and pravastatin were purchased from Sigma-Aldrich (St. Louis, MO, USA). Prior to use, simvastatin needs to be activated by the opening of the lactone ring. For this purpose, we used the protocol provided by Merck. Briefly, eight milligrams of simvastatin (0.019 mM) was dissolved in 0.2 mL of 100% ethanol, with subsequent addition of 0.3 mL of 0.1 N NaOH. The solution was heated at 50 °C for 2 h and then neutralized with HCl to pH 7.2 [16].

### 2.3. MTT Assay

The MTT assay was performed to evaluate the cytotoxicity of statins, as previously described [17]. 3-(4,5-dimethylthiazol-2-yl)-2,5-diphenyltetrazolium bromide (MTT) (Thermo Fisher Scientific) is reduced to formazan by NAD(P)H-dependent oxidoreductase enzymes of viable cells. Briefly, tumor cell lines were treated with the indicated concentrations of statins in DMEM containing 1% FBS. One hour before the end of the incubation period, the supernatants were removed and MTT diluted in DMEM was added. MTT was then removed, and cells were lysed using DMSO for the measurement of the OD at 570 nm using an Infinite M200 PRO (Tecan, Mannedorf, Switzerland) spectrometer.

### 2.4. LDH Assay

LDH release was quantified using a CyQuant LDH Cytotoxicity assay (Thermo Fisher Scientific). The level of formazan is directly proportional to the amount of LDH released in the extracellular milieu and can be detected by reading the absorbance at 490 nm and 680 nm. To determine LDH activity, the 680 nm absorbance value (background) was subtracted from the 490 nm absorbance. The results are presented as fold increased in LDH levels compared to control cells.

### 2.5. Caspase-3 Activity

HepG2 cells were seeded into 12-well plates and were treated with the reported concentrations of simvastatin and pravastatin for 24 h or 48 h. After the incubation period, cells were lysed in caspase buffer containing 50 mM HEPES, pH 7.4 and 0.1% CHAPS. Protein estimates in each sample were determined by performing a BCA protein assay using a BCA Protein Assay Kit (Thermo Fisher Scientific) and following the provided protocol considering BSA as a protein concentration standard. Sample volumes were equilibrated accordingly and transferred into a 96-well plate for the caspase activity assay. The caspase activity assay was performed in a final volume of 200 µL containing the caspase-3 fluorogenic substrate Z-DEVD-AMC (AAT Bioquest, Pleasanton, CA, USA) at final concentration of 50 µM. The plate was then read using a multi-mode plate reader (CLARIOstar Plus, BGM LABTECH, Ortenberg, Germany) at Ex/Em = 355/460 nm.

### 2.6. Bright-Field Microscopy Images

Images showing the changes in cellular conformation after treatment with simvastatin and pravastatin were captured using a Leica Eclipse Ti inverted microscope (Wetzlar, Germany).

### 2.7. Fluorescence Assay

Briefly, cells were cultured overnight in an 8-chamber glass slide (Thermo Fisher Scientific) and later treated with the reported concentration of simvastatin and pravastatin for 24 h or 48 h. Afterwards, cells were washed with PBS and fixed using 4% paraformaldehyde for 30 min. Slides were blocked in 2% BSA, permeabilized with PBS containing 0.1% Triton X-100, stained with Gold Antifade Mountant with DAPI (Thermo Fisher Scientific) and analyzed with a Zeiss Axio Observer Z1 (Carl Zeiss, Oberkochen, Germany) with a 63× oil immersion lens. Pictures analysis was performed using Image J software version 2.14.0/1.54f (National Institutes of Health, Bethesda, MD, USA).

### 2.8. Gene Expression Analysis

RNA was extracted from PC-3, HeLa, HCT116 and HepG2 cell lines using the TRIZOL lysis technique and according to a previously established protocol [18].

RNA was then converted to cDNA using a High-Capacity cDNA Reverse Transcription Kit (Thermo Fisher Scientific). Real-time PCR was performed using the QuantStudio Real-Time PCR System (Thermo Fisher Scientific) with the SYBR green reaction mixture (Thermo Fisher Scientific). The RNA concentration was evaluated using a Nano drop device (Thermo Fisher Scientific). Standardization was performed with house-keeping gene alpha-tubulin. Primers were designed using Primer Blast tool and supplied by Nano-Diagnostika (Vilnius, Lithuania). The primer sequence was Alpha Tubulin (FW-GTGTGGATTCTGTGGAAGGC; RV-ATGAAAGCACACATTGCCAC) and SLOC1B3 (FW-GTCACCTTGTCTAGCAGGATGC; RV-GCATTCACCCAAGTGTGCTGAG).

### 2.9. Protein Expression Profile

The expression profile for the protein of interests was determined using the Human Protein Atlas (HPA) as a database.

### 2.10. Statistical Analysis

Data are presented as arithmetic means ± s.e.m. of at least 3 independent experiments. Statistical values were calculated, and graphs were plotted using Prism version 10.2.0. Data were analyzed using a one-way ANOVA. A Dunnett multiple comparisons test was sued to compare each treatment vs. control. Significance was ascribed at *p* ≤ 0.05 with * *p* ≤ 0.05, ** *p* ≤ 0.01 and *** *p* ≤ 0.001.

## 3. Results

### 3.1. Statins Used in the Study and Differences in Their Uptake

**Figure 1:**
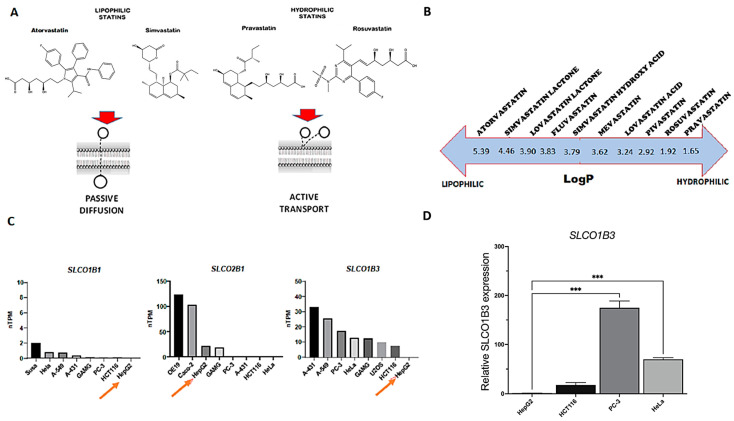
**Different mechanisms of statins to enter cells.** (**A**) Lipophilic statins like simvastatin enter cells mainly by passive diffusion through the membrane bilayer, while hydrophilic statins like pravastatin use active transport by specific transporters. Statin structure, drawn using ChemDraw15.1. (**B**) Statins’ lipophilicity, and hence their ability to cross the cell membrane by passive diffusion, is expressed by logP (octanol/water partition coefficient). For this study, we selected simvastatin acid (logP 3.79) and pravastatin (logP 1.65). (**C**) Genetic expression of pravastatin’s main transporters in tumor cell lines according to the Human Protein Atlas; in hepatocellular carcinoma cells HepG2, pravastatin transporters are poorly expressed. (**D**) *SLCO1B3* gene expression analysis in four cell lines (HepG2, HCT116, HeLa and PC-3). In the graph, the relative expression of each cell line is compared to HepG2 levels (one-way ANOVA and Dunnett’s post hoc test). Standardization was performed with the house-keeping gene alpha-tubulin. Data are presented as arithmetic means (SEM) (n = 3). *** *p* ≤ 0.001.

The hydrophilic or lipophilic nature of statins can influence the entrance into cells and the overall effect on cellular proliferation. Lipophilic statins mainly enter the cells via passive diffusion through the membrane bilayer, while hydrophilic statins require an active transport mechanism (Figure 1A). To determine the effects of statins’ chemical properties on cellular uptake, we selected two statins with different lipophilicities: lipophilic simvastatin hydroxic acid (logP of 3.79 according to Chemaxon), which is the active metabolite of simvastatin lactone, and hydrophilic pravastatin (logP of 1.65 according to Chemaxon). LogP is the most used parameter to indicate the lipophilicity of a compound [19]. The logP of different types of statins is shown in Figure 1B. Pravastatin is a substrate of organic anion transporters OATP1B1 (encoded by the gene *SLCO1B1*), OATP1B3 (encoded by the gene *SLCO1B3*) and OATP2B1 (encoded by the gene *SLCO2B1*) [20,21,22], which are poorly expressed in HCC cell line HepG2 (Figure 1C, orange arrows) according to the Human Protein Atlas database, suggesting a potential deficit of uptake in this cell line. To validate the poor expression of these transporters in our HCC cells, we performed gene analysis of *SLCO1B3* and confirmed the low expression levels compared to other immortalized cell lines (Figure 1D). Indeed, recent clinical data have confirmed that variations in pravastatin pharmacokinetics can be related to the presence of polymorphisms in the genes encoding for organic anion transporters, especially *SLCO1B1* [23,24]. In particular, individuals carrying the 521C allele either in combination with the 388A allele (SLCO1B1*5) or the 388G allele (SLCO1B1*15) exhibited higher mean pravastatin area under the curve (AUC) values compared to individuals carrying reference alleles SLCO1B1*1a or *1b, indicating a decreased pravastatin transport function in the 521C variant [25].

### 3.2. Lipophilic Simvastatin but Not Hydrophilic Pravastatin Influences Cell Cytotoxicity

**Figure 2:**
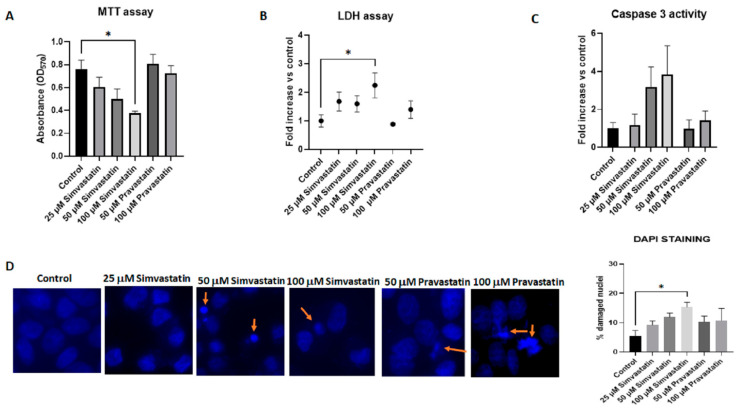
**Lipophilic simvastatin but not hydrophilic pravastatin influences cell viability and apoptosis after 24 h of treatment.** (**A**) HepG2 cells were treated for 24 h with the reported concentration of simvastatin or pravastatin. Viable cells were evaluated by the MTT assay, reading the absorbance at 570 nm. Data are presented as means (SEM) of absorbance values from three independent experiments. Statistical significance was determined using a one-way ANOVA with Dunnett’s post hoc test (comparing each treatment vs. control). (**B**) Leakage of lactate dehydrogenase (LDH) into supernatants was evaluated after 24 h of treatment with the indicated concentrations of statins. Data, presented as fold increases in LDH levels vs. control, are the means (SEM) from three independent experiments. Statistical significance was determined using a one-way ANOVA with Dunnett’s post hoc test (comparing each treatment vs. control). (**C**) Caspase-3 activity in HepG2 cells treated with the indicated concentrations of statins. The assay for caspase-3 was performed using the fluorogenic substrate DEVD-AMC and data are reported as fold increases vs. control. Data are presented as the means (SEM) of four independent experiments and analyzed using ANOVA and Dunnett’s post hoc test. (**D**) HepG2 cells were cultured overnight in 8-well chamber slides in a complete medium. The following day, the complete medium was replaced with DMEM 1% FBS, and cells were treated with the reported concentration of statins for 24 h. After treatment, cells were washed with PBS and fixed in 4% paraformaldehyde for 30 min. The slides were rehydrated in PBS, blocked with a solution containing 2% BSA and permeabilized using 0.1% Triton X-100 solution before addition of DAPI. A Zeiss Axio Observer Z1 was used to analyze nuclear morphological changes: a total of at least 250 cells per treatment were scored for the presence of nuclear changes such as nuclear fragmented bodies and condensed or deformed nuclei (orange arrows). Data are presented as the arithmetic means (SEM) of three experiments, and statistical analysis was performed using a one-way ANOVA and Dunnett’s post hoc test. * *p* ≤ 0.05.

**Figure 3:**
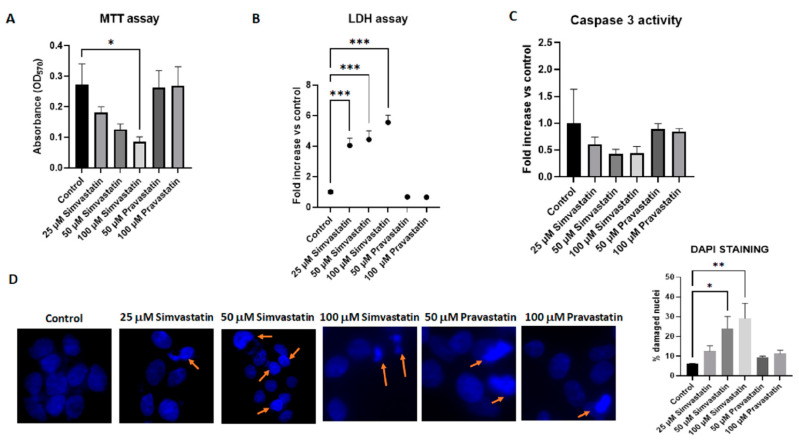
**Lipophilic simvastatin but not hydrophilic pravastatin influences cell viability and apoptosis after 48 h of treatment.** (**A**) HepG2 cells were treated for 48 h with the reported concentration of simvastatin or pravastatin. Viable cells were evaluated by an MTT assay, reading the absorbance at 570 nm. Data are presented as means (SEM) of absorbance values from three independent experiments. Statistical significance was determined using a one-way ANOVA with Dunnett’s post hoc test (comparing each treatment vs. control). (**B**) Leakage of lactate dehydrogenase (LDH) into supernatants was evaluated after 48 h of treatment with the indicated concentrations of statins. Data, presented as fold increases in LDH levels vs. control, are the means (SEM) from four independent experiments. Statistical significance was determined using a one-way ANOVA with Dunnett’s post hoc test. (**C**) Caspase-3 activity in HepG2 cells treated with the indicated concentration of statins. The assay for caspase-3 was performed using the fluorogenic substrate DEVD-AMC (excitation 355 nm, emission 460 nm) and data are reported as fold increases vs. control. Data are presented as the means (SEM) of three independent experiments and were analyzed using an ANOVA and Dunnett’s post hoc test. (**D**) HepG2 cells were treated for 48 h in 8-well chamber slides with the reported concentrations of statins. After treatment, cells were washed with PBS and fixed in 4% paraformaldehyde for 30 min. The slides were rehydrated in PBS, permeabilized and blocked with a solution containing 0.1% triton and 1% bovine serum albumin (BSA) before addition of DAPI. A Zeiss Axio Observer Z1 was used to analyze nuclear morphological changes: a total of at least 300 cells per treatment were scored for the presence of nuclear changes such as nuclear fragmented bodies and condensed or deformed nuclei (orange arrows). Data are presented as the arithmetic means (SEM) of four experiments and statistical analysis was performed using one-way ANOVA and Dunnett’s post hoc test. * *p* ≤ 0.05, ** *p* ≤ 0.01 and *** *p* ≤ 0.001.

To analyze cell viability, we used the MTT assay, which detects metabolic activity. MTT is a water-soluble tetrazolium salt, which is converted to insoluble purple formazan via cleavage of the tetrazolium ring by succinate dehydrogenase within the mitochondria [26]. The formazan product is impermeable to cell membranes and therefore it accumulates only in healthy cells. Our experiments showed that after 24 h, simvastatin induces a dose-dependent decrease in cell viability, while no significant effects were induced by pravastatin (Figure 2A). As an additional measure of cell damage leading to cell death, we compared the leakage of the enzyme lactate dehydrogenase (LDH) in the supernatant of simvastatin- and pravastatin-treated cells. Similar results were observed, with the highest concentration of simvastatin (100 μM) inducing a 2.2-fold increase in LDH compared to the control, while pravastatin treatment induced minimal LDH release (Figure 2B). In line with these results were also the results of measurement of caspase-3 as a marker of apoptosis (Figure 2C) and of nuclear morphological evaluation with DAPI (Figure 2D). In both cases, simvastatin induced a dose-dependent increase in caspase-3 activity and in the number of cells with apoptotic features. The longest exposure time (48 h) enhanced the cytotoxicity of simvastatin, as shown by MTT (Figure 3A) and LDH assays (Figure 3B) and DAPI counting (Figure 3D). Indeed, caspase-3 activation was not observed after 48 h of treatment, both in simvastatin- and pravastatin-treated cells, in line with studies where, in the late stages of apoptosis or in cells that successfully completed apoptosis, caspase activity was downregulated (Figure 3C). This can occur through various mechanisms, including the action of endogenous inhibitors of apoptosis (IAPs), proteolytic degradation of activated caspases, or the activation of anti-apoptotic pathways [27,28,29]. These data confirm previous observations where lipophilic statins but not hydrophilic statins induced cytotoxicity and apoptosis induction in a wide range of cancer cells, including breast, ovarian, endometrial and cervical cancers [30,31,32].

### 3.3. Simvastatin Induces Concentration-Dependent Morphological Changes

**Figure 4:**
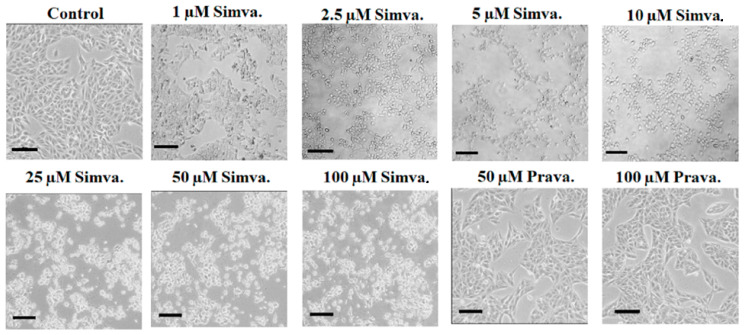
**Effects of simvastatin on the morphology of HepG2 cells.** The morphologies of HepG2 cells changed gradually from a fusiform shape to a round shape after treatment with simvastatin for 24 h (range of concentrations used: 1 μM–100 μM). Morphological changes were observed and captured via an inverted light microscope with a 10× objective (scale bar, 100 μm).

Microscope images of HepG2 cells treated with different concentrations of simvastatin and pravastatin were acquired. After 24 h of treatment, simvastatin induces concentration-dependent shape changes in HepG2 cells as also reported previously for prostate and pancreatic cancer cells [33]. From a fusiform, fibroblast-like morphology, cells acquire a smaller rounded form (Figure 4). However, this effect is present also at concentrations which do not affect cell viability, as confirmed by MTT (Supplementary Figure S1A) and LDH assays (Supplementary Figure S1B), and is related to the induced rearrangement of cytoskeleton proteins [34,35]. No morphological changes were visible after pravastatin treatment at the concentrations used (50 μM and 100 μM). Overall, our results demonstrate that lipophilic simvastatin induced dose- and time-dependent changes in cell viability, while pravastatin, relying on carrier-dependent transports whose expression is downregulated in HepG2 cells, showed limited effects.

### 3.4. Statin Lipophilicty Determines Cytotoxicity in Immortalized Cancer Cells

**Figure 5:**
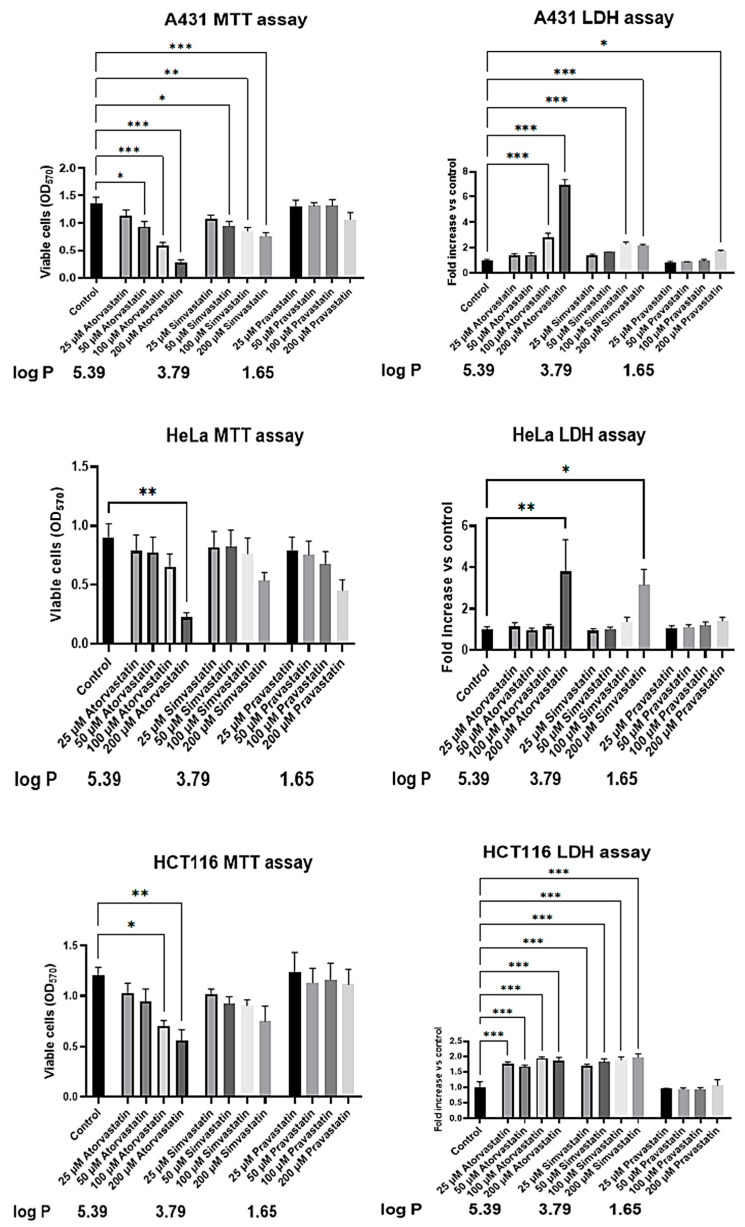
**Statins influence cell viability of tumor cell lines according to their lipophilicity.** A431, HeLa and HCT116 cells were treated for 24 h with the reported concentrations of atorvastatin, simvastatin or pravastatin. Viable cells were evaluated by MTT assays, reading the absorbance at 570 nm. Data are presented as means (SEM) of absorbance values from four (A431, HeLa) or three (HCT116) independent experiments. Statistical significance was determined using a one-way ANOVA with Dunnett’s post hoc test (comparing each treatment vs. control). Leakage of lactate dehydrogenase (LDH) into supernatants was evaluated after 24 h of treatment with the indicated concentrations of statins. Data, presented as fold increases in LDH levels vs. control, are the means (SEM) from four (A431, HeLa) or three (HCT116) independent experiments. Statistical significance was determined using a one-way ANOVA with Dunnett’s post hoc test (comparing each treatment vs. control). * *p* ≤ 0.05, ** *p* ≤ 0.01 and *** *p* ≤ 0.001.

To further prove the importance of lipophilicity in regulating statin cytotoxicity towards cancer cells, we selected four additional immortalized cell lines (A431, HeLa, HCT116 and PC-3). These cell lines express different levels of the *SLCO1B3* gene, which was poorly expressed in HepG2 cells (Figure 1C,D). Furthermore, in our toxicity screening, we added atorvastatin, which has a higher logP (5.39, Chemaxon) and has been extensively used as an adjuvant in cancer therapy [36,37,38]. As shown in Figure 5 left panel (MTT assay) and Figure 5 right panel (LDH assay), atorvastatin is more cytotoxic compared to simvastatin in all tested cells, while pravastatin, due to its hydrophilicity, shows some effects only at the highest concentration used (Figure 5 and Supplementary Figure S2).

These data confirm that lipophilic statins, due to their higher cytotoxic potential, may be beneficial in cancer treatment.

## 4. Discussion

The lipophilicity of drugs plays a significant role in their cytotoxicity, particularly in terms of their ability to penetrate cell membranes and reach their molecular targets within cells [19]. The lipophilicity of statins has a relevant clinical importance due to the fact that, while hydrophilic statins are excreted largely unchanged, lipophilic statins undergo oxidative bio-transformations and therefore are also susceptible to drug–drug interactions [39,40]. Statins possess pleiotropic effects independent of their lipid-lowering properties that may explain the beneficial effects observed for cardiovascular diseases, inflammation and cancer [41,42,43]. However, it is difficult in clinical trials to distinguish between cholesterol-dependent and independent effects, because statins effectively reduce cholesterol levels even in individuals with low baseline cholesterol levels [44]. In connection with cancer, statins’ cytotoxic effects have been observed in multiple human tumor cell lines, including glioma, neuroblastoma and lung and breast cancer cells [33,45,46,47]. In the case of HCC, which accounts for more than 90% of primary liver cancer, statins may enhance the efficacy of immune checkpoint inhibitors (ICIs) through the regulation of inflammatory responses and the immune microenvironment [48,49]. Indeed, the continuous change in the treatment landscape for HCC currently points at a combination of ICIs and tyrosine kinase inhibitors (TKIs) as an effective treatment due to the antiangiogenic and immunomodulatory properties of TKIs [50]. Several studies have highlighted the beneficial effects of using statins in combination with TKIs, particularly to overcome cases of resistance to TKIs, and, among statins, lipophilic simvastatin appeared to be the most effective in different types of tumors [51,52,53]. In our study, simvastatin, but not pravastatin, inhibited the proliferation of HepG2 cells in a dose- and time-dependent manner. Furthermore, simvastatin induced changes in cellular shape at all the concentrations used. Similarly to this study, only lipophilic, but not hydrophilic, statins have been shown to reduce proliferation in colorectal cancer, breast cancer and thyroid cancer cells [54]. The reason behind these observations is that hydrophilic pravastatin exclusively relies on transport carries such as OATP1B1, which is normally expressed in hepatocytes [39,55] but is heavily downregulated in different tumors, including colorectal and liver cancer cells [56,57]. Indeed, depending on their chemical structure, statins are likely to show different intracellular effects. The activation of caspase-3 observed with simvastatin is not only related to the higher transmembrane uptake [30] but also to the increased translocation of Fas into lipid rafts [58]. Selection of additional cell lines (A431, HeLa, HCT116 and PC-3) expressing higher values of organic anion transporters and their addition in our analysis of atorvastatin, with the highest logP (5.39), confirmed that the cytotoxicity is strictly connected to lipophilicity and in general with the ability of statins to enter the cells by passive diffusion [1,59]. Our findings are strengthened by the use of a combinatory approach, where the MTT assay provides information on the metabolic state of cells, while the LDH assay provides insights into membrane damage leading to LDH extracellular leaking. In both assays, a correlation between statin lipophilicity and cytotoxicity can be clearly observed, with atorvastatin inducing in all cases the highest decrease in cell viability. The anti-tumor effects of atorvastatin in HCC have been attributed to several mechanisms, including inhibition of MYC oncogene and protein kinase B (AKT) and induction of senescence [60,61,62]. Furthermore, several studies have provided evidence that the use of lipophilic statins is also a potential protective factor against the development of HCC [63,64,65]. Selection of statins according to their logP value is an important factor to consider, since their cytotoxicity, while undesirable in simple hyperlipidemia therapy, could have beneficial effects in cancer prevention and therapy.

## 5. Conclusions

In this study, variations in statin lipophilicity (indicated by the logP value) affect the cytotoxic potential in HCC cells and other tumor cell lines of different origins. We validated our findings using viability assays measuring the metabolic state of the cells (MTT) and the membrane integrity (LDH release). Our results suggest that the application of statins in cancer therapy is strictly connected to the evaluation of their physicochemical properties, which include factors such as lipophilicity, solubility and molecular structure. These properties influence how statins interact with cellular membranes and to what extent they accumulate within cancer cells.

## Data Availability

All data are provided in the main text.

## Supplementary Materials

The following supporting information can be downloaded at https://www.mdpi.com/article/10.3390/biology13060455/s1: Figure S1: (A) HepG2 cells were treated for 24 h with the reported concentration of simvastatin (range of concentrations used: 1 μm–100 μM) and pravastatin (concentrations used: 50 μm and 100 μM). Viable cells were evaluated by MTT assays, reading the absorbance at 570 nm. Data are presented as means (SEM) of absorbance values from five independent experiments. Statistical significance was determined using a one-way ANOVA with Dunnett’s post hoc test (comparing each treatment vs. control). (B) HepG2 cells were treated for 24 h with the reported concentration of simvastatin (range of concentrations used: 1 μm–100 μM) and pravastatin (concentrations used: 50 μm and 100 μM). Leakage of lactate dehydrogenase (LDH) into supernatants was evaluated using a CyQuant LDH Cytotoxicity assay. Data are presented as means (SEM) of fold increases in LDH levels versus untreated cells (n = 5). Figure S2: (A) PC-3 cells were treated for 48 h with the reported concentrations of atorvastatin, simvastatin or pravastatin. Viable cells were evaluated by MTT assays, reading the absorbance at 570 nm. Data are presented as means (SEM) of absorbance values from three independent experiments. Statistical significance was determined using a one-way ANOVA with Dunnett’s post hoc test (comparing each treatment vs. control). (B) PC-3 cells were treated for 48 h with the reported concentrations of atorvastatin, simvastatin or pravastatin. Leakage of lactate dehydrogenase (LDH) into supernatants was evaluated using a CyQuant LDH Cytotoxicity assay. Data are presented as means (SEM) of fold increases in LDH levels versus untreated cells (n = 3).

## Abbreviations

BCA	Bicinchoninic acid
DAPI	4′,6-diamidino-2-phenylindole
DMEM	Dulbecco’s Modified Eagle Medium
DMSO	Dimethyl sulfoxide
FBS	Fetal bovine serum
HCC	Hepatocellular carcinoma
HMG-CoA	β-Hydroxy β-methylglutaryl-CoA
IAP	Inhibitor of apoptosis protein
ICI	Immuno-checkpoint inhibitor
IU	International unit
LDH	Lactate dehydrogenase
LDL	Low-density lipoprotein
MTT	3-(4,5-dimethylthiazol-2-yl)-2,5-diphenyl-2H-tetrazolium bromide
OATP1B1	Organic-anion-transporting polypeptide 1B1
OATP1B3	Organic-anion-transporting polypeptide 1B3
OATP2B1	Organic-anion-transporting polypeptide 2B1
OD	Optical density
SEM	Standard error of the mean
*SLCO1B1*	Solute Carrier Organic Anion Transporter Family Member 1B1
*SLCO2B1*	Solute Carrier Organic Anion Transporter Family Member 2B1
*SLCO1B3*	Solute Carrier Organic Anion Transporter Family Member 1B3
TKI	Tyrosine kinase inhibitor
