# Supplementary material for: Effect of Statin Lipophilicity on the Proliferation of Hepatocellular Carcinoma Cells
Source: Biology (Basel). 2024 Jun 19;13(6):455. doi: 10.3390/biology13060455 (PMC11200858; doi:10.3390/biology13060455)
Supplement: Supplementary file 1 [file biology-13-00455-s001.zip › biology-2985870-supplementary.pdf]

biology

MDPI

Article

Effect of Statin Lipophilicity on the Proliferation of Hepatocellular Carcinoma Cells

Goda Glebavičiūtė, Akshay Kumar Vijaya and Giulio Preta

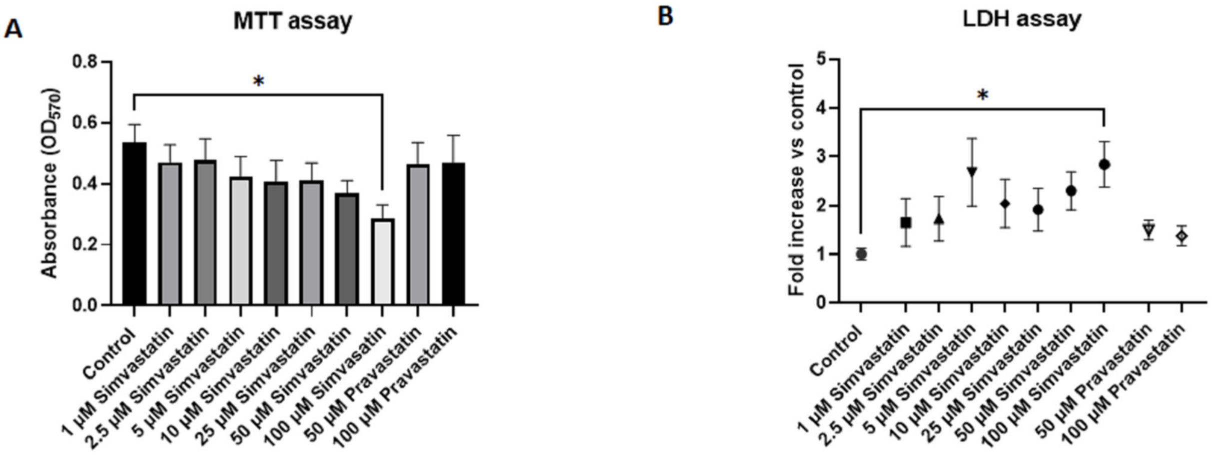

Figure S1. A) HepG2 cells were treated for 24 h with the reported concentration of simvastatin (range of concentrations used 1 µm-100 µM) and pravastatin (concentrations used 50 µm and 100 µM). Viable cells were evaluated by MTT assay, reading the absorbance at 570 nm. Data are presented as mean (SEM) of absorbance values from five independent experiments. Statistical significance is determined using one-way ANOVA with Dunnett's post hoc test (comparing each treatment vs control). B) HepG2 cells were treated for 24h with the reported concentration of simvastatin (range of concentrations used 1 µm-100 µM) and pravastatin (concentrations used 50 µm and 100 µM). Leakage of lactate dehydrogenase (LDH) into supernatants was evaluated using CyQuant LDH Cytotoxicity assay. Data are presented as mean (SEM) fold increase in LDH levels versus untreated cells (n=5).

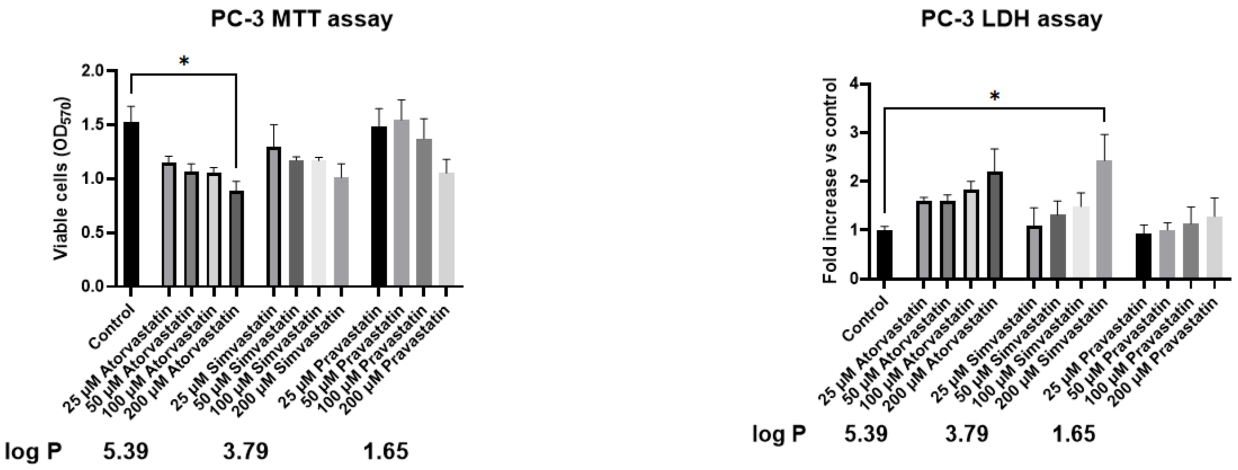

Figure S2. PC-3 cells were treated for 48 h with the reported concentrations of atorvastatin, simvastatin or pravastatin. Viable cells were evaluated by MTT assay (left panel), reading the absorbance at 570 nm. Data are presented as mean (SEM) of absorbance values from three independent experiments. Statistical significance is determined using one-way ANOVA with Dunnett's post hoc test (comparing each treatment vs control). Leakage of lactate dehydrogenase (LDH) into supernatants was evaluated using CyQuant LDH Cytotoxicity assay (right panel). Data are presented as mean (SEM) fold increase in LDH levels versus untreated cells (n=3).
